# A Vision-Guided Point Cloud Refinement Method for Helical Surface Measurement: A Case Study on Screw Thread Pitch

**DOI:** 10.3390/s26185976

**Published:** 2026-09-21

**Authors:** Weimin Lou, Huan Chen, Peng Yang, Jian Wu, Ying Zhou, Kaiqing Chen, Lu Liu, Ming Kong, Yuanhai Jiang, Weixin Ruan, Xinlei Zhang

**Affiliations:** 1 Zhejiang Key Laboratory of Digital Precision Measurement Technology Research, Zhejiang Institute of Quality Sciences, Hangzhou 310018, China; lwmoptics@zju.edu.cn (W.L.); bro_young@foxmail.com (P.Y.); zjimkfb@163.com (J.W.); 15888808261@163.com (Y.Z.); chenkaiqing233@163.com (K.C.); 2 College of Metrology Measurement and Instrument, China Jiliang University, Hangzhou 311222, China; lu_liu@cjlu.edu.cn (L.L.); mkong@cjlu.edu.cn (M.K.); 3 College of Civil Engineering and Architecture, Jiaxing University, Jiaxing 314200, China; szjyh888@163.com; 4 Zhejiang Chenxin Machine Equipments Co., Ltd., Shaoxing 312300, China; zjchenxinjx@163.com; 5 Tiezheng Testing Technology Co., Ltd., Chongqing 405800, China; 18785395999@163.com

**Keywords:** line-structured laser scanning, vision-guided point cloud refinement, screw thread pitch measurement, 2D-3D registration

## Abstract

Screws are critical mechanical components, and their geometric accuracy directly determines the performance and reliability of mechanical systems. However, conventional contact-based inspection methods are time-consuming and limited to sparse sampling. Existing non-contact optical techniques often suffer from insufficient edge localization accuracy due to discrete point spacing. To address these challenges, this paper proposes a vision-guided point cloud refinement measurement method. The system integrates a laser scanner with a camera, where the laser scanning serves as the primary channel for acquiring three-dimensional point clouds, and the vision system provides high-resolution thread edge positions through sub-pixel localization and interpolation. A joint 2D-3D registration procedure is established to transfer the 2D sub-pixel edge localization precision to the corresponding 3D points, effectively correcting the spatial positions of thread crests. A coarse-to-fine processing pipeline is then applied, encompassing PCA-based principal axis alignment, Fourier transform-based coarse pitch estimation, and Gaussian pyramid-based multi-resolution refinement, to extract the pitch from the refined point cloud. Experimental validation on a real screw specimen confirms that the proposed method achieves a pitch measurement error of 0.0423 mm, demonstrating performance comparable to the pure vision-based method. It can provide a reliable solution for high-precision screw thread inspection.

## 1. Introduction

Screw threads are ubiquitous mechanical components that serve critical functions in fastening, power transmission, motion conversion, and sealing across virtually all industrial sectors, including aerospace, automotive, energy, and heavy machinery. Their geometric accuracy directly determines the functional performance and operational reliability of mechanical systems [1,2,3]. As manufacturing tolerances continue to tighten, the precision inspection of helical surfaces has become a critical yet challenging task in modern quality assurance.

Traditional inspection methods for screws are predominantly based on contact-type coordinate measuring machines (CMMs) or specialized thread measuring instruments [4,5]. While these approaches offer high measurement accuracy, they suffer from inherent limitations: time-consuming point-by-point probing, probe wear issue, and sparse sampling strategies that cannot capture the full three-dimensional morphology of complex helical surfaces [6]. Moreover, the conventional approach evaluates thread parameters from only two axial cross-sections, which inherently fails to capture three-dimensional geometric anomalies. These limitations underscore an urgent need for measurement techniques capable of acquiring and analyzing full-field three-dimensional geometric data of helical surfaces.

Recent advances in three-dimensional thread metrology have demonstrated the feasibility of holistic evaluation approaches based on areal measurement strategies. Researchers have developed comprehensive frameworks encompassing mathematical parameterization of screw thread geometry, areal data acquisition using modern coordinate measuring technology, and three-dimensional point cloud evaluation [7]. The novel 3D approach enables separate probing of thread flanks, allowing form deviations to be assigned to individual flanks and providing insight into the location and causes of manufacturing errors. A unified theory for helical machine elements has also been proposed, introducing a universal 3D geometry model with distinct parameterization using a unique set of geometry parameters [8]. These studies confirm the validity of point-cloud-based approaches for thread metrology with high reproducibility. However, these methods were developed primarily for tactile CMM systems and have not been fully extended to optical line laser scanning scenarios, where point cloud characteristics differ substantially.

Non-contact optical measurement technologies have emerged as promising alternatives to address the limitations of contact-based inspection. Among these, line-structured light (line laser) scanning has gained particular attention due to its high acquisition speed, high spatial resolution, active illumination that reduces sensitivity to surface reflectivity variations, and seamless integration into automated inspection workflows [9]. Line-scan cameras enable in-motion three-dimensional reconstruction under evident motion changes, making them suitable for inline quality inspection and defect detection [10]. Monocular structured light-based systems have demonstrated that generated point clouds can quantify defects with millimeter-level accuracy [11]. In gear inspection, line structured light technology offers a rapid, non-contact solution with a maximum deviation of only 4 μm compared to contact methods [9], and coarse-to-fine methods have been established for extracting geometric information from structured light images [12]. To further enhance the measurement accuracy of thread profiles, sub-pixel edge detection techniques have been developed, which achieve high accuracy and good stability for the positioning of sub-pixel edge coordinates [13].

Despite these advantages, line-structured light systems are susceptible to measurement errors when scanning highly reflective surfaces. Multiple reflections and specular highlights can corrupt the captured laser stripe images, leading to inaccurate centerline extraction and consequently producing outlier points and data voids in the reconstructed point cloud [14]. To enable accurate fusion of laser and vision data, extrinsic calibration between the two sensors is a prerequisite. Recent reviews have systematically summarized the state of the art in this field. Qiu et al. provided a comprehensive review of external multi-modal imaging sensor calibration for sensor fusion, examining various characteristics and conditions of different calibration methods [15]. Tan et al. further reviewed deep learning-based approaches for LiDAR and camera extrinsic calibration, covering both accurate calibration estimation and relative calibration prediction [16]. Robust preprocessing methods are therefore essential. Additionally, acquired point clouds typically contain hundreds of thousands or millions of points, imposing significant computational burdens and potentially propagating errors [17].

Consequently, point cloud data reduction has become an essential preprocessing step [18]. Curvature-based approaches utilizing k-neighbor searching and principal component analysis effectively preserve point cloud features while ensuring uniform distribution [19], and KD-tree-guided algorithms have shown significant improvements in feature-point retention and PSNR [20]. However, many existing methods lack systematic validation mechanisms, and critical geometric features may be lost during simplification [21].

Beyond data reduction, extracting geometric parameters from helical surface point clouds presents additional challenges. Helical geometries are characterized by continuous curvature variations and three-dimensional periodicity, making crest and root identification a challenging task. Direct extremum detection is susceptible to sampling errors, as measured points rarely coincide with true extremum positions. Surface reconstruction has been extensively studied using both parametric and non-parametric approaches [22], with non-linear least squares fitting of Bézier surfaces [23], least squares B-spline approximation for irregularly scattered point clouds [24], and deep learning-assisted B-spline parameterization [25] all contributing to the field. However, global surface fitting demands substantial computational resources and may suffer from accuracy degradation due to over-smoothing or under-fitting. A strategy combining coarse localization with refined local fitting is therefore highly desirable.

The coarse-to-fine strategy has also been successfully applied to other structured surfaces. For instance, for structured surfaces such as corrugated plates, a non-contact depth detection method integrating a line laser sensor with a precision linear guide has been developed, enabling continuous acquisition of high-resolution surface profiles. A multi-stage data reduction strategy combining angle-chord height criteria with spline-based filtering was proposed, followed by a two-stage feature recognition algorithm: coarse analysis locates candidate peaks and valleys, followed by fine detection using local B-spline fitting [26]. The success of this coarse-to-fine paradigm for two-dimensional profiles suggests that a similar approach can be extended to three-dimensional helical surfaces.

To address the aforementioned challenges, this paper proposes a line-structured laser scanning-based measurement method. There are two contributions of this paper. The primary one is the extraction of sub-pixel edge positions from camera images. Then these positions are used to directly correct the spatial coordinates of thread crest points in the laser point cloud. It transfers the high lateral resolution of machine vision directly to the 3D measurement domain. Additionally, a systematic coarse-to-fine processing pipeline is established. It contains PCA-based principal axis alignment, Fourier transform-based coarse pitch estimation, and Gaussian pyramid-based multi-resolution refinement. The combination of these techniques, in which the Fourier transform provides a noise-robust initial estimate and the Gaussian pyramid enables sub-sampling crest localization, constitutes a methodological contribution that addresses the specific challenges of thread metrology from point cloud data. The laser scanning system serves as the primary measurement channel, acquiring dense three-dimensional point clouds, while high-resolution 2D thread edge positions extracted from camera images are registered with the point cloud through a joint 2D-3D correspondence procedure. This enables the sub-pixel localization precision of the vision channel to be transferred to the corresponding 3D points, effectively correcting the spatial positions of thread crests and improving the accuracy of subsequent pitch measurement. The proposed method thus combines the full-field 3D measurement capability of laser scanning with the high-resolution edge localization capability of machine vision, achieving enhanced measurement precision while maintaining comprehensive spatial coverage. The effectiveness of the proposed approach is validated through comparative experiments on both simulated and real thread point cloud data.

## 2. Methodology

**Figure 1:**
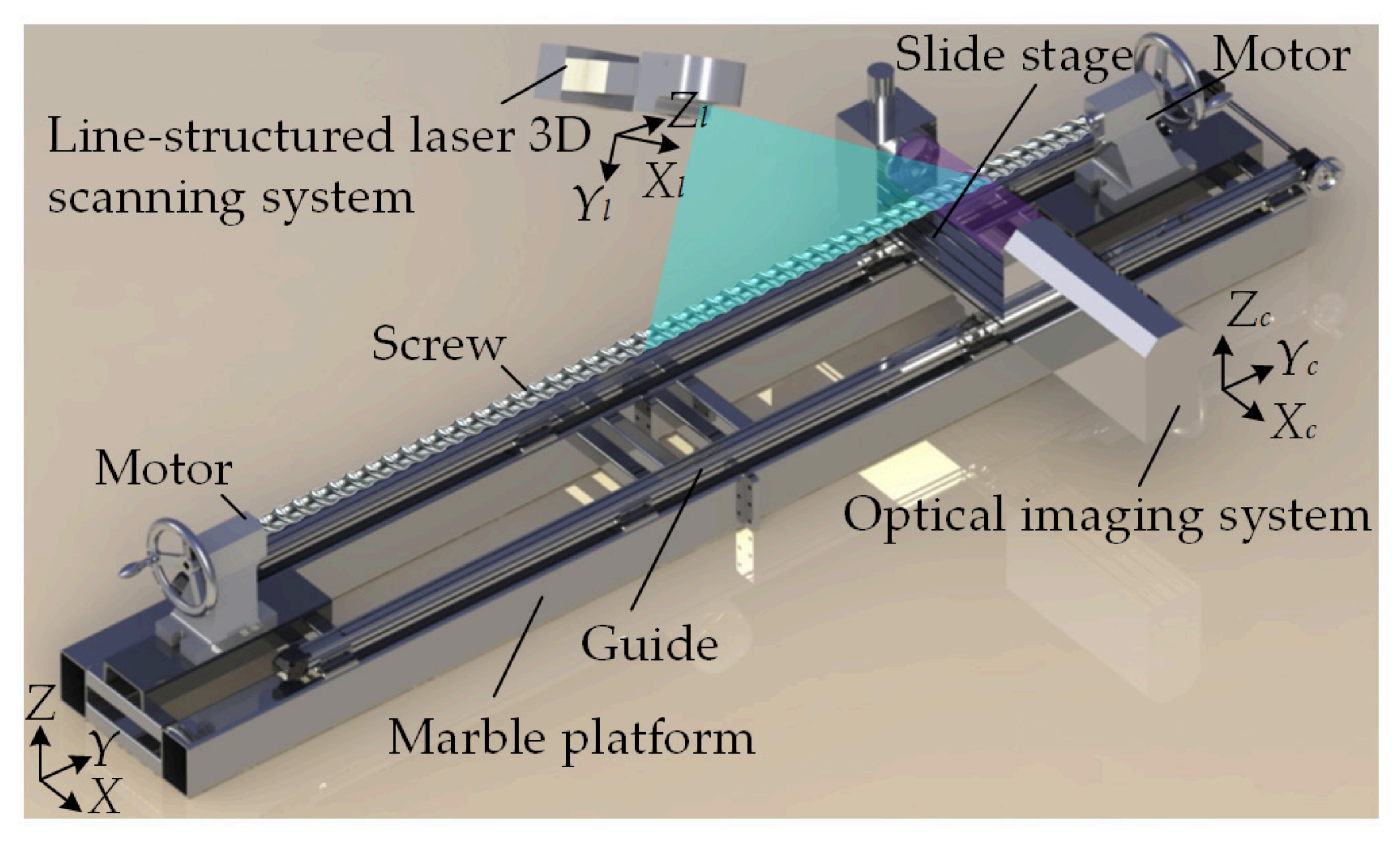
Schematic diagram of the measurement system.

The system configuration developed in this paper is shown in Figure 1, which is primarily composed of three main modules: a marble platform, a clamping and motor driving unit, and a scanning and imaging unit. The measured screw is securely clamped between two centers at both ends and is driven to rotate about its axis by rotary motors. The optical imaging system is mounted on a precision linear guide rail, enabling it to perform a scanning motion along the axial direction of the screw thread. Meanwhile, the line-structured laser 3D scanning system is above the screw and operates in coordination with the optical imaging system to continuously acquire complete surface profile data along the axial direction.

The measurement principle of the proposed system is as follows. The line-structured laser projector emits multiple laser stripes on the screw surface. The deformation of these stripes, as captured by the laser scanner’s built-in camera, encodes the three-dimensional profile of the thread surface based on the laser triangulation principle. Simultaneously, the optical imaging system acquires high-resolution two-dimensional images of the thread profile under backlight illumination, providing clear edge information of the thread crests and roots. These two sensing modalities operate in a complementary manner. The laser scanner provides full-field 3D surface data with reliable depth information, while the vision system offers superior lateral resolution for precise edge localization. The raw data from both sensors are then fused through a joint 2D-3D registration procedure, where the sub-pixel edge positions extracted from the vision images are used to refine the corresponding 3D points in the laser point cloud. This synergistic integration enables the system to achieve both comprehensive spatial coverage and high measurement accuracy for screw thread inspection.

**Figure 2:**
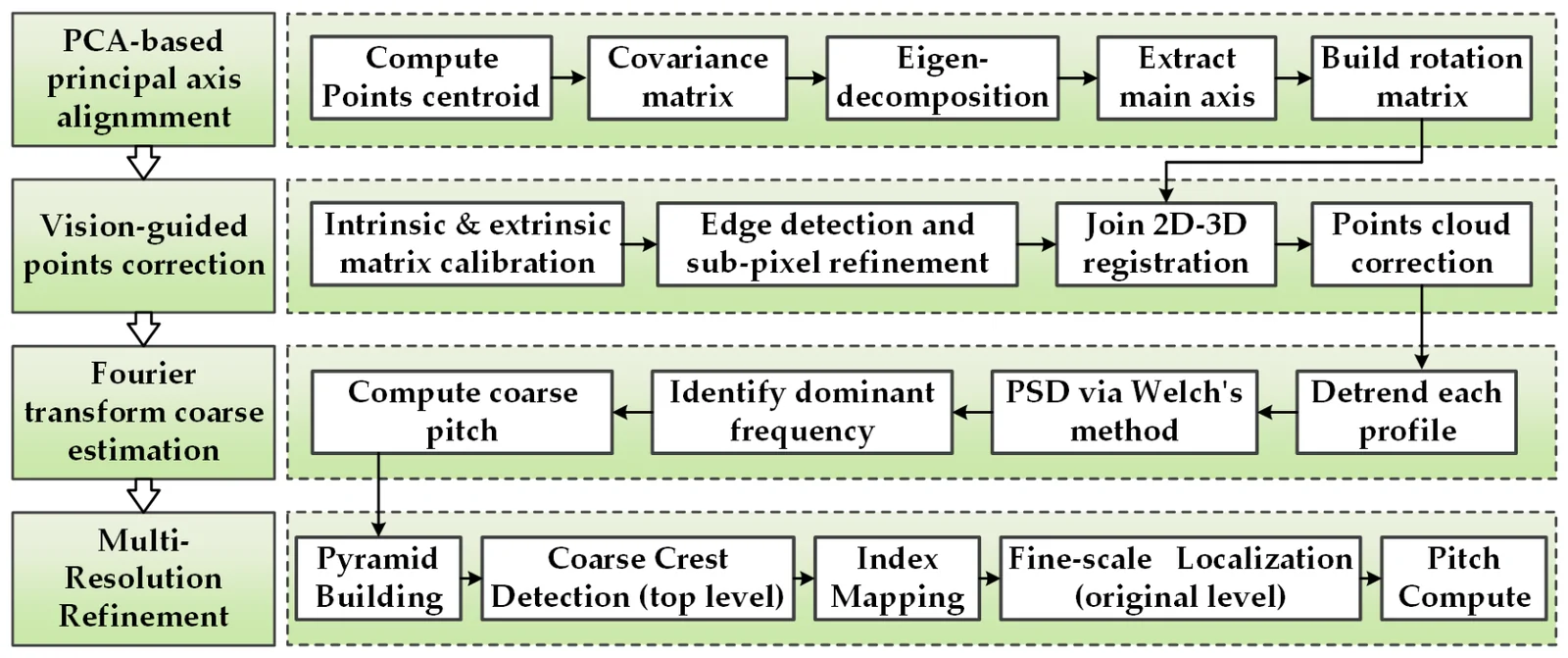
Flowchart of point cloud data processing.

The overall laser point cloud data processing pipeline consists of four sequential stages as illustrated in Figure 2: (1) PCA-based principal axis alignment for eliminating spatial pose deviations; (2) Vision-guided point cloud correction through intrinsic/extrinsic calibration, sub-pixel edge refinement, and joint 2D-3D registration; (3) Fourier transform-based coarse pitch estimation from multi-section profiles for obtaining a global initial value; and (4) Gaussian pyramid-based multi-resolution refinement guided by the coarse estimate for sub-sampling crest localization and accurate pitch measurement.

### 2.1. Point Cloud Principal Axis Alignment by PCA Method

In practical measurement scenarios, the fixture posture of the screw thread workpiece can hardly guarantee strict parallelism between its geometric axis and the coordinate axes of the measurement system, resulting in inevitable spatial pose deviations in the acquired point cloud data. Directly extracting cross-sectional profiles or performing feature analysis along any fixed coordinate axis of the measurement frame would introduce systematic geometric errors, thereby compromising the measurement accuracy of subsequent geometric parameters such as pitch and thread profile. Consequently, it is essential to accurately estimate the principal axis orientation of the point cloud prior to further processing, and to transform the entire point cloud into a canonical coordinate system aligned with the estimated principal axis.

In this study, the Principal Component Analysis (PCA) method is adopted to estimate the axial direction of the screw thread point cloud. For a slender cylindrical point cloud distribution characteristic of screw threads, the direction of maximum variance inherently corresponds to the geometric axis of the screw thread.

The centered point cloud data can be expressed as,

(1) $${P^{\prime }}_{i}=P_{i}-\bar{P}=P_{i}-\frac{1}{N}\sum_{i=1}^{N}P_{i}$$

where $P_{i}={\left[x_{i},y_{i},z_{i}\right]}^{T}(i=1,2,\ldots ,N)$ is the coordinate matrix of the point cloud and $\bar{P}$ is the centroid matrix. The covariance matrix C, which characterizes the spatial dispersion of the point cloud along different directions, is constructed as:

(2) $$C=\frac{1}{N}\sum_{i=1}^{N}\left[(P_{i}-\bar{P}\right){\left(P_{i}-\bar{P}\right)}^{T}]$$

Its eigenvectors and corresponding eigenvalues are obtained by solving the characteristic equation:

(3) $$Cv_{j}=\lambda_{j}v_{j},(j=1,2,3)$$

where $\lambda_{1},\lambda_{2},\lambda_{3}$ are the eigenvalues sorted in descending order and $v_{1},v_{2},v_{3}$ are the corresponding orthonormal eigenvectors. The first principal component $v_{1}$, associated with the largest eigenvalue $\lambda_{1}$, represents the direction of maximum variance and is taken as the estimated principal axis of the point cloud. Finally, we can transform the centered point cloud data ${P^{\prime }}_{i}$ to the new point cloud data ${P^{\prime \prime }}_{i}$ with point cloud principal axis alignment by Equation (4).

(4) $$\begin{array}{c} {P^{\prime \prime }}_{i}=R\cdot P^{\prime }\\ \left\{\begin{array}{c} R={\left[{e^{\prime }}_{x},{e^{\prime }}_{y},{e^{\prime }}_{z}\right]}^{T}\\ {e^{\prime }}_{x}=(v_{1}\times e_{z})/|v_{1}\times e_{z}|\\ {e^{\prime }}_{y}=v_{1}\times {e^{\prime }}_{x}\\ {e^{\prime }}_{z}=v_{1} \end{array}\right. \end{array}$$

where $e_{z}={\left[0,0,1\right]}^{T}$.

### 2.2. Camera Calibration

Although the laser point cloud-based method after principal axis alignment by the PCA method provides comprehensive three-dimensional surface information, the localization accuracy of thread crests is inherently limited by the discrete point spacing of the acquired point cloud. As a result, the measured crest positions may deviate from their true physical locations by up to half the point spacing, which introduces systematic errors in subsequent pitch calculations. To overcome this limitation, a vision-guided point cloud refinement strategy is introduced. The vision system, operating as an auxiliary module, provides high-resolution two-dimensional thread edge positions with sub-pixel accuracy. Through a joint 2D-3D registration procedure, these precise edge locations are transferred to the corresponding points in the laser point cloud, enabling correction of the spatial positions of crest points and thereby improving the accuracy of pitch measurement.

Camera calibration was performed based on a planar checkerboard pattern with known grid spacing. The calibration procedure yields the intrinsic parameter matrix $K_{\mathrm{in}}$ and the lens distortion coefficients. After distortion correction, the spatial resolution of the imaging system is determined by imaging a calibration target of known physical dimensions.

Additionally, the rotation matrix $R_{\mathrm{out}}$ and translation matrix $T_{\mathrm{out}}$ between the camera and the laser scanner are determined through a joint calibration procedure using a custom-designed calibration target visible to both sensors. These parameters are essential for establishing accurate 2D-3D correspondences in the subsequent registration step.

### 2.3. Sub-Pixel Edge Localization and Interpolation Algorithm

The sub-pixel edge localization and interpolation algorithm consists of two phases as depicted in Algorithm 1. In the first phase, pixel-level edges are detected using the Canny operator, and a quadratic polynomial is fitted along the gradient direction of each edge point. The sub-pixel edge position is determined as the extremum of the fitted parabola. In the second phase, the detected edge points are filtered using median and Gaussian smoothing to remove noise. After outlier rejection based on residual analysis, the dominant frequency is extracted via FFT, and a cosine-based interpolation is performed to generate the final high-density profile with sub-pixel accuracy.


**Algorithm 1** **Sub-Pixel Edge Localization and Interpolation Algorithm****Input:** Gray image I(u,v), number of interpolation points $N_{f}$ and outlier rejection threshold η. **Output:** Edge profile with sub-pixel accuracy ${\left\{(u_{j}^{fine},v_{j}^{fine})\right\}}_{j=1}^{N_{f}}$. **Phase 1: Image-domain sub-pixel edge localization** 1. Apply Gaussian smoothing to I(u,v) for suppressing high-frequency noise and obtain $I_{smooth}(u,v)$; 2. Detect pixel-level edges using the Canny operator: $\xi_{pix}\leftarrow Canny(I_{smooth})$, where $\xi_{pix}={\left\{P_{k}\right\}}_{k=1}^{M}$ and $P_{k}=(u_{k},v_{k})$; 3. **for** each pixel-level edge point $\left(u_{k},v_{k}\right)$ **do** 4. Compute the gradient direction $n_{k}=\nabla I_{smooth}(u_{k},v_{k})/\left|\nabla I_{smooth}(u_{k},v_{k})\right|$; 5. Extract the intensity profile along $n_{k}$ and fit a quadratic polynomial $f(x)=ax^{2}+bx+c$ to the profile; 6.      **if** a<0 7.          $x_{peak}\leftarrow -0.5\cdot b/a$ and $P_{k}^{sub}\leftarrow P_{k}+x_{peak}\cdot n_{k}$; 8.      **else** 9.          $P_{k}^{sub}\leftarrow P_{k}$; 10.    **end if** 11. **end for** **Phase 2: Interpolation along the edge** 12. Apply median filter to $P_{k}^{sub}=(u_{k}^{sub},v_{k}^{sub})$ for removing impulsive noise and get $P_{k}^{med}$; 13. Apply Gaussian smoothing to $P_{k}^{med}=(u_{k}^{med},v_{k}^{med})$ for suppressing high-frequency noise and get $P_{k}^{smooth}=(u_{k}^{smooth},v_{k}^{smooth})$; 14. Compute the residual $\varepsilon_{k}=v_{k}^{smooth}-v_{k}^{sub}$; 15. Compute residual standard deviation $\sigma =\sqrt{\frac{1}{N-1}\sum_{k=1}^{N}{\left(\varepsilon_{k}-\bar{\varepsilon }\right)}^{2}}$ and let *t* = 0 for counting; 16.    **for** *k* = 1 **to** *N* **do** 17.        **if** $\varepsilon_{k}>\eta \cdot \sigma$ then 18.              t←t+1, mark $\left(u_{k}^{sub},v_{k}^{sub}\right)$ as an outlier and remove; 19.        **end if** 20.    **end for** 21.    N←N−t and determine the dominant frequency $f_{peak}$ by FFT-based pitch estimation; 22. Extract the phase φ of the dominant frequency component from the FFT result; 23. Compute the mean radius $\bar{v}=\frac{1}{N}\sum_{k=1}^{N}v_{k}$ and amplitude $A=0.5\cdot [\max(v_{k})-\min(v_{k})]$; 24. **for** *j* = 1 **to** $N_{f}$ **do** 25.        $u_{j}^{fine}\leftarrow u_{1}+\frac{j-1}{N_{f}-1}(u_{N}-u_{1})$ and $v_{j}^{fine}\leftarrow \bar{v}+A\cdot \cos[2\pi f_{peak}\cdot (u_{j}^{fine}-u_{1})+\varphi ]$; 26. **end for** 27. return ${\left\{(u_{j}^{fine},v_{j}^{fine})\right\}}_{j=1}^{N_{f}}$;


The basic numerical setting parameters are listed in Table 1. The Canny thresholds were set to 50 (low) and 170 (high). A 5 × 5 Gaussian filter was applied to the image. The edge contour was processed by a 1 × 11 median filter and a 1 × 7 Gaussian filter. The outlier rejection threshold was set to 3.

### 2.4. Vision-Guided Point Cloud Correction by Joint 2D-3D Registration

To transfer the high-resolution edge information from the image domain to the laser point cloud, a joint 2D-3D registration procedure is performed. Using the pre-calibrated extrinsic parameters, each 3D point is projected onto the image plane:

(5) $${z^{\prime }}_{i}{\left[u_{i},v_{i},1\right]}^{T}=K_{\mathrm{in}}(R_{\mathrm{out}}P_{i}+T_{\mathrm{out}})$$

where ${z^{\prime }}_{i}$ is the depth scale factor and $P_{i}={\left[x_{i},y_{i},z_{i}\right]}^{T}$ is the coordinate vector of the *i*-th 3D point. The projection yields a set of 2D image coordinates $Q_{i}={\left[u_{i},v_{i}\right]}^{T}$ for each 3D point. The detected sub-pixel edge points are denoted as $G_{j}={\left[{u^{\prime }}_{j},{v^{\prime }}_{j}\right]}^{T}$. A nearest-neighbor search is then performed to establish correspondence between the edge points and the projected 3D points. For each edge point $G_{j}$, the closest projected point $Q_{i^{*}}$ within a search radius *r* (3 pixels) is identified:

(6) $$i*=\arg\underset{i\in ℑ}{\min}{\left\|G_{j}-Q_{i}\right\|}_{2},(ℑ=\left\{i\left|{\left\|G_{j}-Q_{i}\right\|}_{2}<r\right.\right\})$$

For each established correspondence, the sub-pixel edge position $G_{j}={\left[{u^{\prime }}_{j},{v^{\prime }}_{j}\right]}^{T}$ from the vision system is used to correct the spatial position of the corresponding 3D point. The 3D point $P_{i*}={\left[x_{i*},y_{i*},z_{i*}\right]}^{T}$ is first transformed to the camera coordinate system and expressed as $R_{\mathrm{out}}P_{i*}+T_{\mathrm{out}}={\left[x_{i*}^{c},y_{i*}^{c},z_{i*}^{c}\right]}^{T}$, where $z_{i*}^{c}$ is the depth value retained from the original laser measurement, as the line-structured laser 3D scanning system provides reliable depth information. The lateral coordinates are then recomputed using the sub-pixel edge position:

(7) $$\left\{\begin{array}{c} x_{i*}^{ce}=({u^{\prime }}_{j}-u_{0})z_{i*}^{c}/f_{x}\\ y_{i*}^{ce}=({v^{\prime }}_{j}-v_{0})z_{i*}^{c}/f_{y} \end{array}\right.$$

where $\left(u_{0},v_{0}\right)$ are the principal point coordinates and $f_{x},f_{y}$ are the focal lengths in pixels from the intrinsic calibration. The corrected point ${P^{\prime }}_{i*}$ of original point $P_{i*}$ can be finally obtained as:

(8) $${P^{\prime }}_{i*}=R_{\mathrm{out}}^{-1}({\left[x_{i*}^{ce},y_{i*}^{ce},z_{i*}^{c}\right]}^{T}-T_{\mathrm{out}})$$

This correction effectively transfers the sub-pixel localization precision of the vision channel to the corresponding 3D point, improving the spatial accuracy of crest points in the point cloud. The corrected point cloud is obtained by replacing the original points with their corrected counterparts.

Several strategies are adopted to handle imperfect data. When a 3D point is occluded, no sub-pixel edge point falls within the search radius. The correspondence is then discarded, and the original point is retained. For multiple matches, only the closest projected point is selected for each edge point, and if several edge points compete for the same 3D point, the one with the smallest distance wins. Each 3D point is corrected at most once. To suppress false correspondences, two checks are applied. The search radius of 3 pixels serves as a distance threshold, and after correction the reprojection error is verified. In addition, corrections that exceed 1 pixel are rejected. Only matches that pass both checks contribute to the refined point cloud.

The corrected point cloud, with its crest positions refined to sub-pixel-equivalent accuracy, serves as the input for the subsequent pitch extraction procedures. As will be detailed in Section 2.5 and Section 2.6, this refined point cloud undergoes Fourier transform-based coarse estimation followed by Gaussian pyramid-based multi-resolution refinement to obtain the final pitch measurement. The vision-guided correction thus provides a high-quality data foundation that enhances the reliability and accuracy of the subsequent frequency-domain and multi-scale analyses.

### 2.5. Thread Pitch Coarse Estimation Based on Fourier Transform

After principal axis alignment and vision-guided point cloud correction, the thread point cloud is transformed into a canonical coordinate system with the *Z*-axis precisely aligned with the thread axis. During this process, sub-pixel edge information extracted from the vision system is projected back into 3D space through joint 2D-3D registration, effectively correcting the discretization errors of the original laser point cloud at the crest regions. Then multiple longitudinal section profiles $R_{k}(z),(k=1,2,\ldots ,n)$ are extracted from the aligned point cloud at different circumferential angles. Each profile represents the radial variation of the thread surface along the axial direction and exhibits a distinct periodic pattern corresponding to the thread pitch.

Accurate determination of thread pitch from such profiles is a critical step in the measurement pipeline. However, direct peak detection in the spatial domain is susceptible to measurement noise, local surface irregularities, and discretization errors, often leading to unstable or biased results. Moreover, the presence of harmonic distortions or non-uniform sampling along the *Z*-axis may further compromise the reliability of direct spatial methods. To obtain a robust initial estimate of the pitch, a frequency-domain analysis is performed prior to the subsequent fine-scale localization.

In this study, the Fourier Transform is employed to extract the dominant spatial frequency component from each section profile. For a given profile R(z), the fundamental periodicity corresponds to the thread pitch *p*, and its reciprocal defines the spatial frequency *f* = 1/*p*. By transforming the profile from the spatial domain into the frequency domain via the Fast Fourier Transform (FFT), the dominant periodic component can be identified as the peak in the power spectral density (PSD).

To eliminate low-frequency trends caused by potential taper or slight bending of the threaded workpiece, each profile is first detrended by subtracting a linear least-squares fit. Let $z_{i}$ and $R(z_{i})$ denote the axial coordinate and radius value of the *i*-th sampled point in a section profile, respectively. The detrended signal $R_{d}(z_{i})$ is computed as:

(9) $$R_{d}(z_{i})=R(z_{i})-(\alpha z_{i}+\beta )$$

where *α* and *β* are coefficients obtained by linear regression. This operation removes the linear component while preserving the periodic fluctuations that are essential for pitch estimation.

To satisfy the uniform sampling requirement of the FFT and Welch’s methods, the detrended profile is then resampled onto a uniformly spaced Z-grid using linear interpolation. The original Z-coordinates are non-uniformly distributed due to the random sampling nature of the point cloud acquisition. The resampling is performed as follows:

(10) $$\begin{array}{l} R_{d}^{u}(z_{j}^{u})=\mathrm{interp}[z_{i},R_{d}(z_{i}),z_{j}^{u}]\\ \left\{\begin{array}{c} z_{j}^{u}=z_{1}+(j-1)\Delta z,j=1,2,\ldots ,N^{\prime }\\ \;\Delta z=\mathrm{mean}[\mathrm{diff}(z_{i})] \end{array}\right. \end{array}$$

where $N^{\prime }$ is the number of points on the uniform grid, interp[]  is a linear interpolation function, diff( ) is a difference function and mean[ ] is an average function. Subsequently, the power spectral density (PSD) of the uniformly resampled signal is estimated using Welch’s averaged periodogram method, which reduces the variance of the spectral estimate by averaging multiple overlapping segments. The spatial sampling frequency is then determined from the uniform interval:

(11) $$f_{s}=1/\Delta z$$

The PSD is then computed and the dominant frequency $f_{peak}$ is identified as the frequency corresponding to the maximum PSD value:

(12) $$f_{\mathrm{peak}}=\underset{f}{\arg\max}PSD(f,f_{s})$$

The coarse pitch estimate $p_{\mathrm{coarse}}$ is then obtained as

(13) $$p_{\mathrm{coarse}}=1/f_{\mathrm{peak}}$$

### 2.6. Thread Pitch Refinement Estimation Based on Multi-Resolution Analysis

The Fourier transform-based method described in Section 2.5 provides a robust global estimate of the thread pitch by identifying the dominant spatial frequency component of the section profiles. However, the frequency-domain estimate is inherently an averaged value over the entire measured length and does not capture local pitch variations along the thread axis. Moreover, direct peak detection on the original profile at full resolution is susceptible to measurement noise and discretization errors, as the sampled points rarely coincide exactly with the true extremum positions. To overcome these limitations and obtain a refined pitch estimate with sub-sampling accuracy, a multi-resolution analysis based on the Gaussian pyramid is proposed.

Let the original profile signal at full resolution be denoted as ${\left\{z_{i}^{\left(0\right)},R^{\left(0\right)}(z_{i}^{\left(0\right)})\right\}}_{i=1}^{N_{0}}$, where $N_{0}$ is the number of sampled points. This signal is defined as Level 0 of the pyramid. For each subsequent level *l* = 1, 2, …, *L*, the signal is constructed by applying Gaussian smoothing followed by dyadic down sampling to the previous level:

(14) $$\left\{\begin{array}{c} R_{\mathrm{smooth}}^{\left(l-1\right)}=R^{\left(l-1\right)}⊗G_{\sigma }\\ R^{\left(l\right)}(z_{i})=R_{\mathrm{smooth}}^{\left(l-1\right)}(z_{2i}) \end{array}\right.$$

where $G_{\sigma }$ is a one-dimensional Gaussian kernel with scale parameter σ, and ⊗ denotes the convolution operation. This process iteratively reduces the signal length by half at each level while preserving the dominant low-frequency components that correspond to the thread periodicity.

To ensure that at least five complete thread periods are preserved at the top level for reliable peak detection, the pyramid level *L* is selected as:

(15) $$L=\max\{2,\min[5,floor(\log_{2}\frac{N_{0}p_{\mathrm{coarse}}/5}{z_{\max}-z_{\min}})]\}$$

where $N_{0}$ is the number of sampled points, $z_{\max}=\max(z_{i}^{\left(0\right)})$ and $z_{\min}=\min(z_{i}^{\left(0\right)})$ at level 0. floor[] is the floor function.

At the coarsest level *L*, the signal is dominated by the fundamental periodic component, while high-frequency noise has been largely suppressed. Crest positions are detected using a sliding window method. The sliding window radius is set to approximately one-quarter of a thread period:

(16) $$w=\max\{3,floor[\frac{N_{L}p_{\mathrm{coarse}}}{4(z_{\max}-z_{\min})}]\}$$

where $N_{L}$ is the number of sampled points at level *L*. Equation (16) ensures that each window covers a sufficiently small fraction of the thread period, guaranteeing that at most one crest is contained within any window while providing adequate local context for extremum detection. A point *i* is identified as a candidate crest if it satisfies two criteria: it is the local maximum within the window, and its value exceeds the mean radius by a prescribed threshold. The detected coarse peak indices at level *L* are denoted as $\kappa_{L}=\{k_{1},k_{2},\ldots ,k_{M_{L}}\}$, where $M_{L}$ is the number of coarse peaks at level *L*. Each coarse peak index $k_{m}$ is then mapped back to the original resolution through successive doubling $k_{m}\cdot 2^{L},(m=1,2,\ldots ,M_{L})$. This mapping provides an initial approximation of the crest positions at the original resolution. At the original resolution, a local neighborhood of radius (typically 15–25 points) is extracted around each mapped index $k_{m}\cdot 2^{L}$. Within each neighborhood, a quadratic polynomial $az^{2}+bz+c$ is fitted to the data points using the least-squares method and the refined crest position $z_{peak,m}$ is obtained by $z_{peak,m}=-0.5b/a$, where a,b,c are the quadratic polynomial coefficients. The retained pitch values are finally averaged to obtain the refined estimate for a single section:

(17) $$\begin{array}{l} p^{ref}=\frac{1}{m_{eff}}\sum_{m=1}^{m_{eff}}p_{m}^{eff}\\ \left\{\begin{array}{c} p_{m}=z_{peak,m+1}-z_{peak,m},(m=1,2,\ldots ,M_{L}-1)\\ p_{m}^{eff}=p_{m},((1-\chi )p_{coarse}\leq p_{m}\leq (1+\chi )p_{coarse}) \end{array}\right. \end{array}$$

where $m_{eff}$ is the number of valid pitch values $p_{m}^{eff}$ after outlier rejection, χ is the outlier rejection coefficient. To obtain a comprehensive measurement that accounts for the full circumferential variation of the thread, the same processing pipeline is applied independently to each of the nine extracted sections (nine longitudinal section profiles are extracted from the aligned point cloud at circumferential intervals of 40°). The final pitch estimate is then computed as the arithmetic mean of the individual section results,

(18) $$\bar{p}=\frac{1}{9}\sum_{k=1}^{9}p_{k}^{ref}$$

where $p_{k}^{ref}$ denotes the refined pitch estimate obtained from the *k*-th section using Equation (17).

## 3. Results and Discussion

### 3.1. Simulation Results and Discussion

To validate the effectiveness of the proposed method, a three-dimensional surface point cloud model of the roller screw was first constructed according to the parameters listed in Table 2, and random Gaussian noise was added to emulate the signal interference encountered in actual measurement scenarios.

#### 3.1.1. Validation of Principal Axis Alignment Accuracy

**Figure 3:**
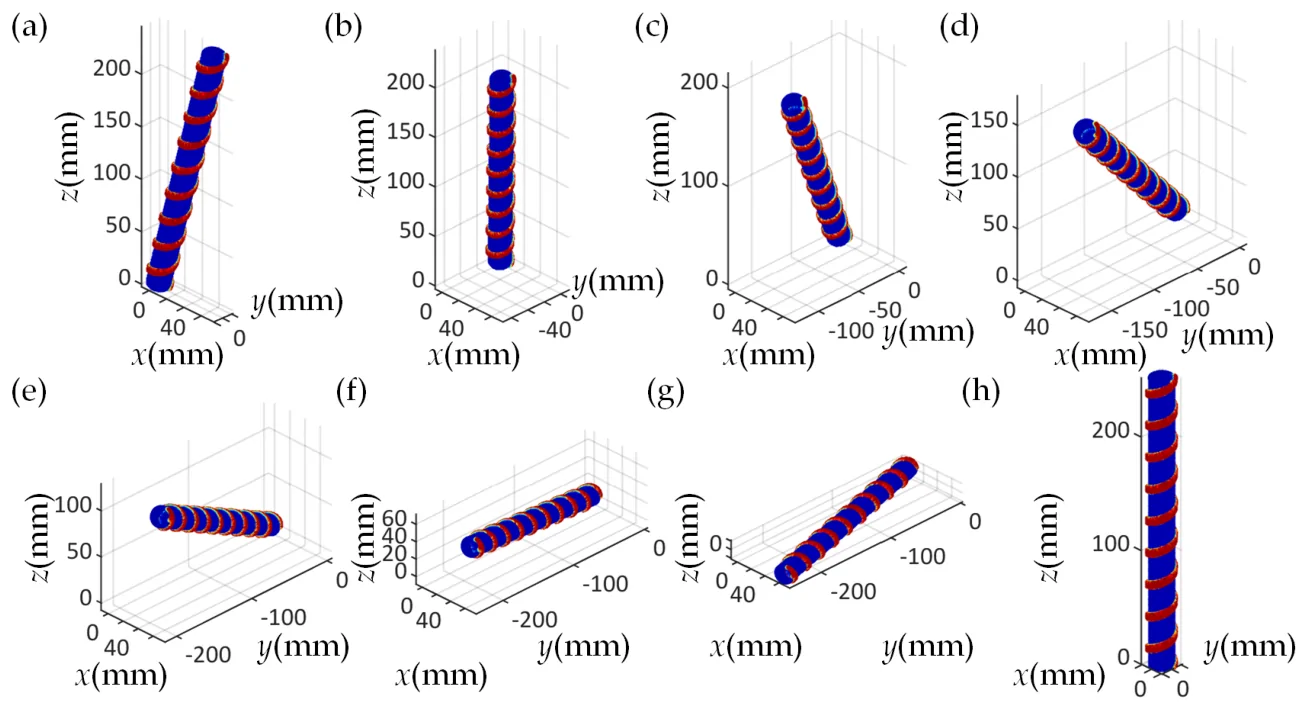
Simulated point clouds of the screw under various spatial orientations. The point clouds are color−coded by radial distance from the axis (blue: root, red: crest). The rotation angles about the *X*− and *Y*−axes are: (**a**) 0°/15°, (**b**) 15°/15°, (**c**) 30°/15°, (**d**) 45°/15°, (**e**) 60°/15°, (**f**) 75°/15°, (**g**) 90°/15°, and (**h**) 0°/0°.

**Figure 4:**
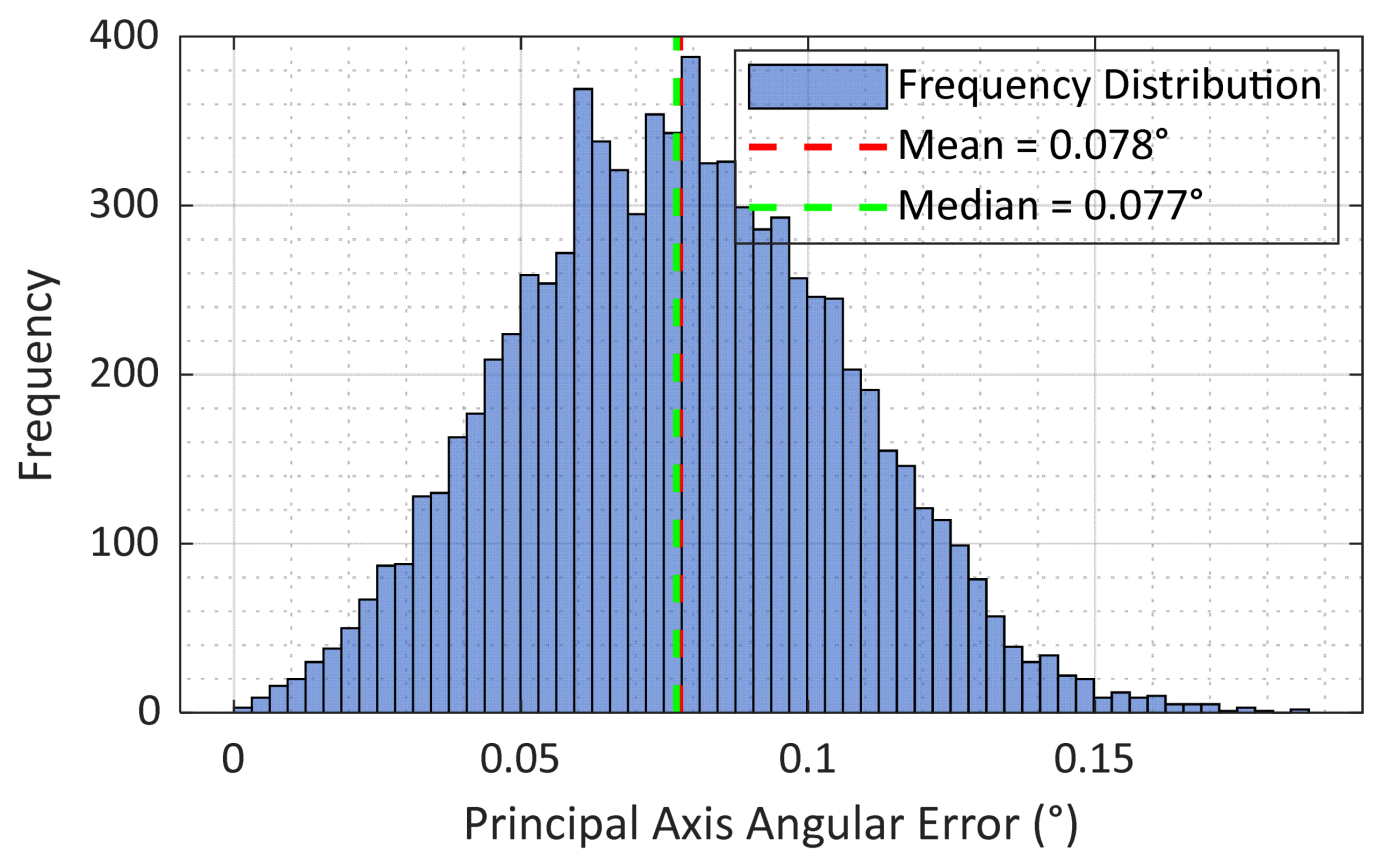
Frequency distribution of PCA principal axis estimation error.

To evaluate the effectiveness of the proposed principal axis alignment method, simulation experiments were conducted on the screw under various spatial orientations. In the initial configuration, the screw axis was aligned with the *Z*-axis. Subsequently, the screw was rotated about both the *X*-axis and *Y*-axis independently, with step increments of 1° from 0° to 90°, generating a total of 8281 pose combinations. Representative alignment results for selected orientations are presented in Figure 3. Figure 4 illustrates the distribution of angular deviations between the corrected axis and the true *Z*-axis after applying the method described in Section 2.1. Statistical analysis reveals that the mean angular error across all 8281 poses is 0.078°.

**Figure 5:**
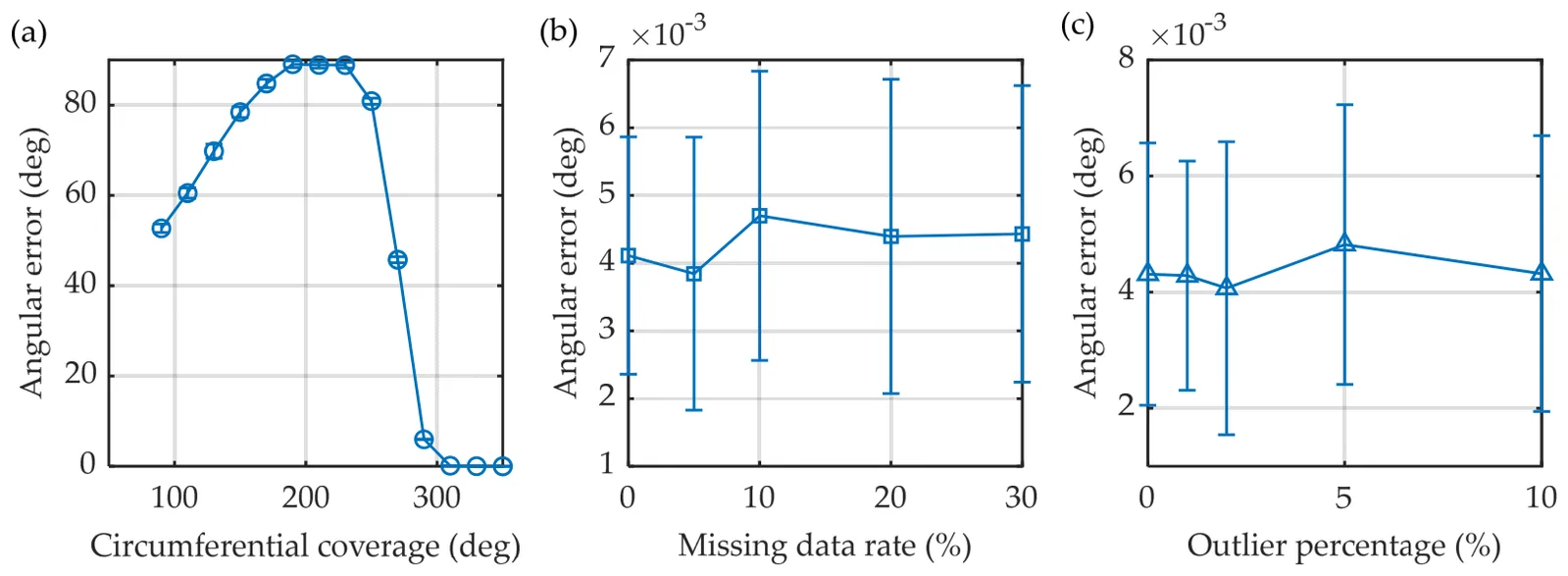
Robustness analysis of PCA principal axis alignment with 50 Monte Carlo runs under a fixed screw orientation (tilt angles of 35° about the *X*-axis and 18° about the *Y*-axis, with an azimuth angle of 0°): (**a**) Partial circumferential coverage, (**b**) Random missing points, (**c**) Outlier contamination.

The robustness of the PCA-based principal axis alignment was evaluated under three non-ideal conditions, and the results are presented in Figure 5. Each data point represents the mean angular error over 50 independent Monte Carlo runs.

As shown in Figure 5a, the angular error is highly sensitive to the circumferential coverage of the point cloud. When the coverage angle is below 270°, the PCA-estimated principal axis deviates severely from the true axis, with the mean error increasing from 5.98° at 290° coverage to 52.72° at 90° coverage. Once the coverage exceeds 310°, the angular error drops sharply, confirming that the PCA alignment requires a sufficiently complete circumferential point distribution to reliably estimate the principal axis.

In contrast, the PCA alignment exhibits remarkable robustness to random missing points and outlier contamination, as shown in Figure 5b,c. For missing data rates ranging from 0% to 30%, the mean angular error remains nearly constant at approximately 0.004°, with standard deviations below 0.003°. Similarly, for outlier percentages from 0% to 10%, the mean angular error stays at approximately 0.004°, with no discernible increasing trend. These results demonstrate that random data loss and sparse outlier contamination have a negligible effect on the principal axis estimation, provided that the overall circumferential coverage remains complete. The insensitivity to outliers can be attributed to the fact that the outliers are randomly distributed in space and their contributions to the covariance matrix are averaged out over the large number of inlier points.

Overall, these results indicate that the PCA-based principal axis alignment is robust to random missing points and outlier contamination but is critically dependent on the circumferential coverage of the point cloud.

#### 3.1.2. Validation of Multi-Resolution Refinement and Error Analysis

**Figure 6:**
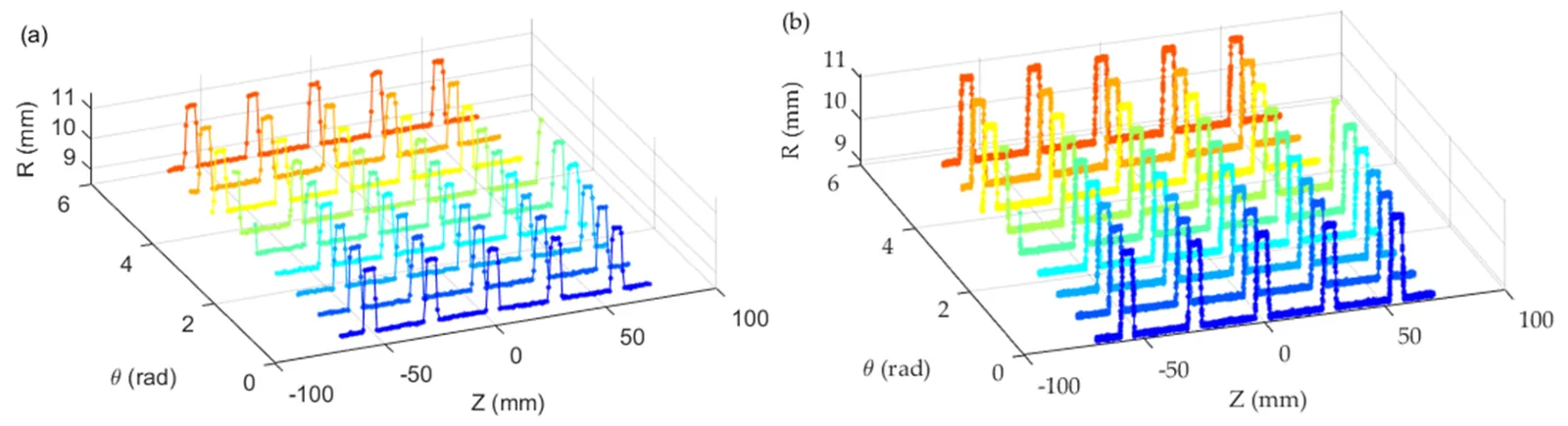
Sub−pixel interpolation of two-dimensional thread contour profiles: (**a**) original discrete contour profiles extracted from circumferentially distributed sections; (**b**) refined contour profiles after sub-pixel interpolation.

**Figure 7:**
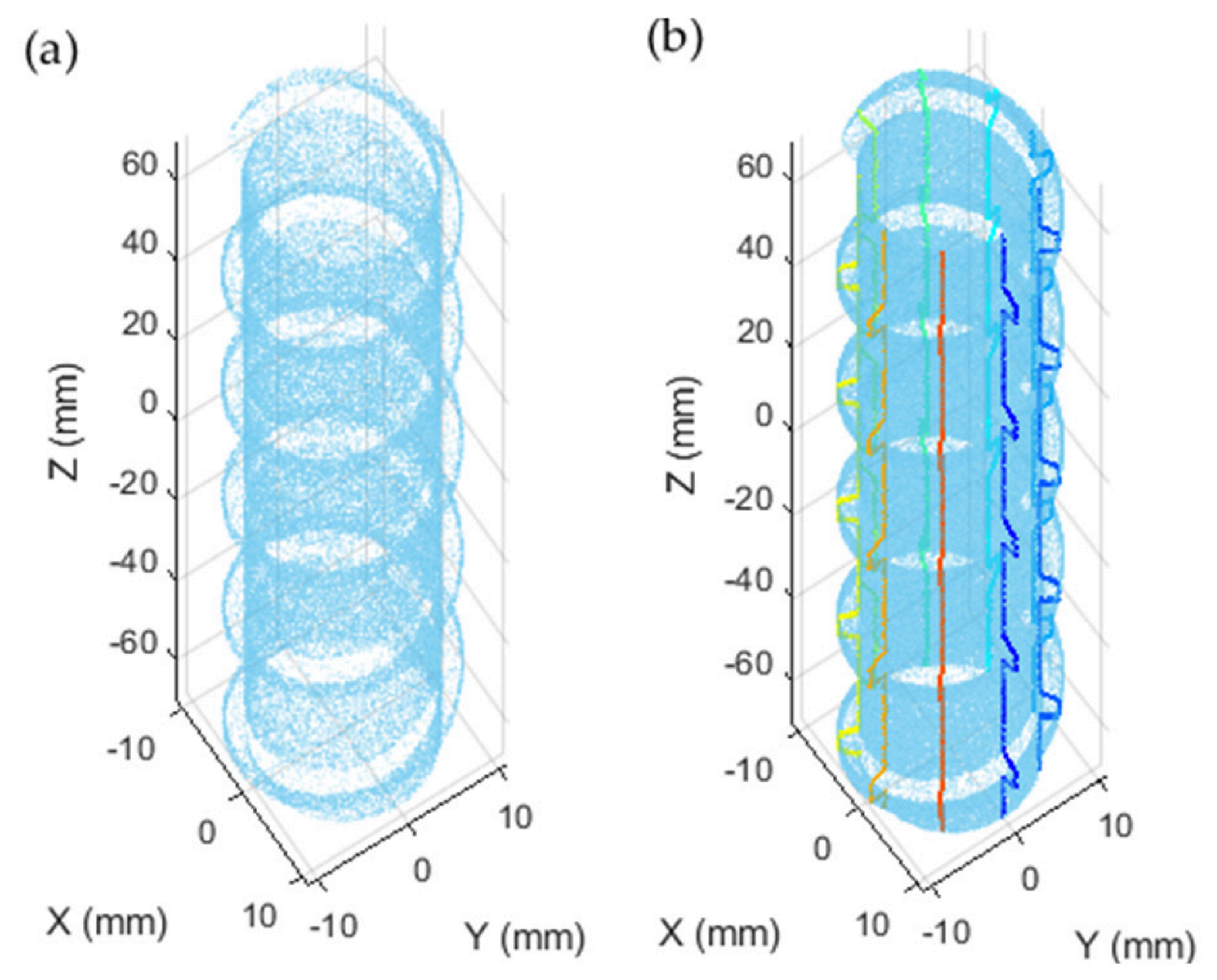
Comparison of three−dimensional point clouds before and after sub−pixel edge refinement: (**a**) original point cloud without sub−pixel interpolation; (**b**) refined point cloud after applying two−dimensional sub−pixel edge interpolation and reprojection.

Figure 6a presents a set of two-dimensional contour profiles acquired at uniformly distributed circumferential positions along the screw thread, which serve as the input for the subsequent sub-pixel interpolation process. Through sub-pixel interpolation, the refined two-dimensional contour profiles are obtained, as shown in Figure 6b. The interpolated contours are then projected back onto the three-dimensional point cloud, yielding the results depicted in Figure 7. Specifically, Figure 7a shows the original point cloud without edge-based sub-pixel interpolation, while Figure 7b presents the refined point cloud after the application of two-dimensional edge sub-pixel interpolation.

**Figure 8:**
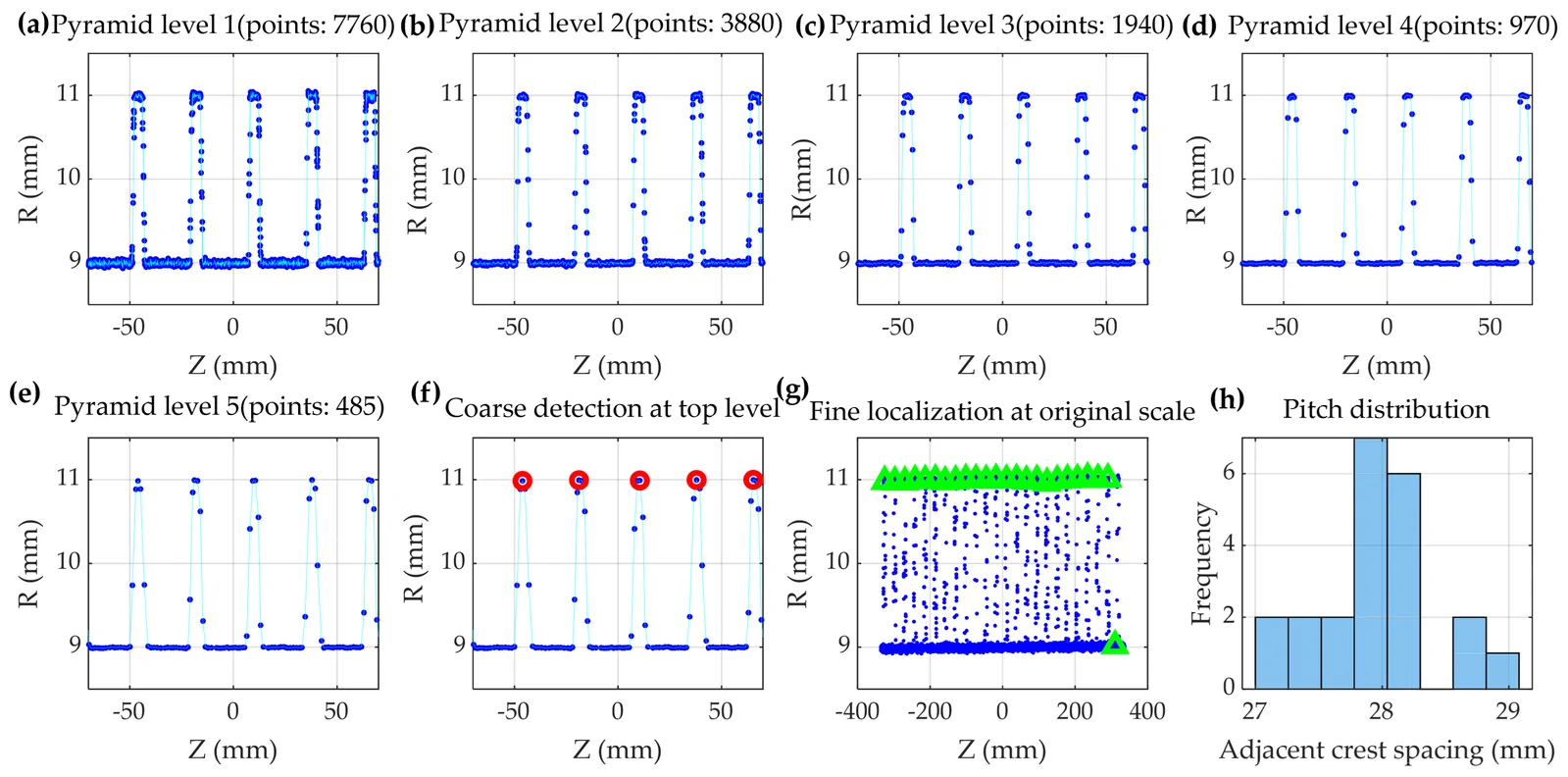
Gaussian pyramid multi−scale pitch extraction simulation results. (**a**–**e**) Pyramid layers 1 to 5 (from original to coarsest scale). (**f**) Coarse crest detection at the top level. (**g**) Fine crest localization at the original scale. (**h**) Histogram of local pitch distribution.

Figure 8 presents the multi-scale pitch extraction simulation results based on Gaussian pyramid analysis, using a screw with a pitch of 28 mm as an illustrative example. It should be noted that the number of pyramid levels is not fixed but is adaptively determined by the algorithm according to the signal length and the initial pitch estimate obtained from the Fourier-based coarse estimation. Specifically, Equation (14) automatically calculates the required number of levels by ensuring that at least five complete thread periods are preserved at the top level, with the result constrained within a range of 2 to 5 levels. For the present dataset, the adaptive calculation yields 5 pyramid levels, comprising eight subfigures: five layers of pyramid waveforms, coarse detection at the top level, fine localization at the original scale, and the pitch distribution histogram.

The pyramid waveforms at each level are shown in Figure 8a–e, corresponding to Levels 1 to 5, respectively. Level 1 represents the original section profile. The original section profile contains 7760 sampled points, which are extracted from the full point cloud (4.74 × 10^5^ points) along a single longitudinal section. As the pyramid level increases, the signal is successively smoothed by Gaussian filtering and downsampled by a factor of two, with the number of points decreasing to 3880, 1940, 970, and 485, respectively.

The coarse detection results at the top level are presented in Figure 8f. At the coarsest scale, consisting of only 485 sampled points, the algorithm successfully identified 24 crest positions, which are marked with red circles. All detected points are accurately located at the actual wave peaks, with no missed or false detections, confirming the reliability of the coarse detection strategy at this scale. Since the signal at the top level has been largely free from high-frequency noise, the local maxima corresponding to the thread crests are distinctly recognizable, demonstrating the robustness of the coarse localization results.

Subsequently, the coarse peak indices were progressively mapped back to the original scale, followed by fine localization through local quadratic polynomial fitting. The refined crest positions, denoted by green triangles, are shown in Figure 8g. When superimposed on the original profile, the refined points are observed to coincide precisely with the crest locations, achieving significantly higher positioning accuracy than the coarse detection results. The pitch estimated from the refined crest positions is 27.967 mm, corresponding to a relative deviation of 0.12% from the true value of 28 mm. This outcome validates the effectiveness of the Gaussian pyramid-based “coarse-to-fine” strategy in enhancing crest localization accuracy.

Figure 8h displays the histogram of pitch distribution after outlier rejection. The valid pitch values are concentrated around 28 mm, indicating good consistency among adjacent crest spacings and confirming the high repeatability of the measurement results.

#### 3.1.3. Sensitivity Analysis

**Figure 9:**
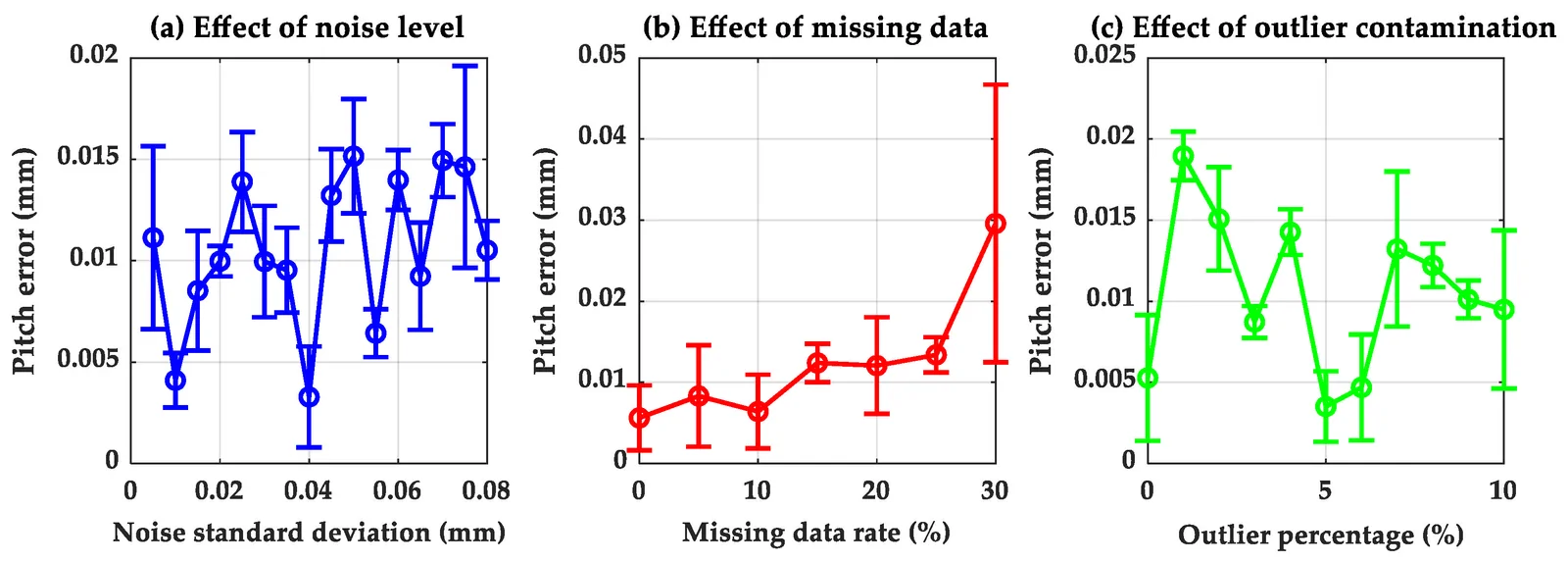
Effect of (**a**) noise level, (**b**) missing data and (**c**) outlier contamination with 50 Monte Carlo runs.

50 independent Monte Carlo runs were conducted as shown in Figure 9. Gaussian noise with standard deviations from 0.005 mm to 0.08 mm was added to the simulated point clouds to evaluate noise tolerance. Figure 9a shows that the mean pitch errors remain below 0.015 mm across the entire range, with no clear increasing trend. Some spikes likely stem from the random spatial distribution of sampled points relative to critical crest regions, rather than a systematic failure of the algorithm. The method therefore handles typical industrial noise levels, with occasional instability under specific noise realizations.

The effect of incomplete data was examined by randomly discarding 5% to 30% of points. As shown in Figure 9b, pitch errors increase from 0.006 mm to 0.03 mm. The increase becomes more pronounced beyond 20% dropout, suggesting that the multi-section strategy can compensate for moderate data loss but has limited resilience when large portions of the surface are absent.

Outlier contamination was evaluated by injecting 1~10% random outliers. Figure 9c shows that pitch errors remain below 0.02 mm across the range, with no increasing trend. The outlier rejection mechanism effectively excludes spurious points, maintaining accuracy even at 10% contamination. Small fluctuations reflect outlier placement variability, but overall performance is stable.

Collectively, these analyses confirm that the proposed method delivers stable pitch measurements under noise and outlier contamination, but is more sensitive to missing data, highlighting the importance of complete surface coverage in practical inspection.

### 3.2. Experimental Results and Discussion

#### 3.2.1. Experimental Setup and Workpiece Specifications

**Figure 10:**
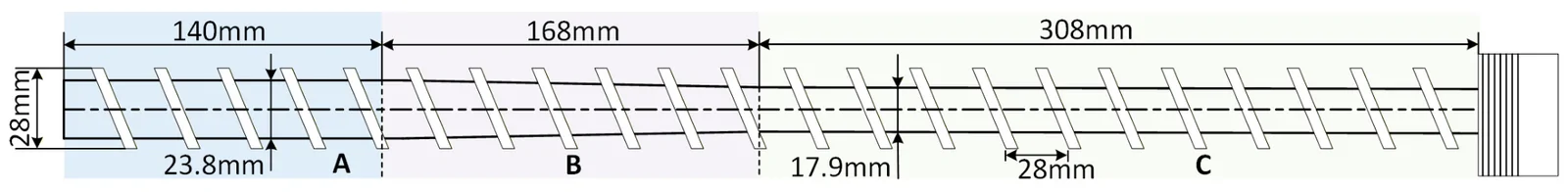
Schematic diagram of the tested screw thread (the root diameter is constant in regions A and C, with A larger than C, while region B serves as a transitional section).

The dimensional drawing of the tested screw thread is shown in Figure 10. According to the root diameter, the screw thread can be divided into three distinct regions. In Region A, the nominal root diameter is 23.8 mm. Region B serves as a transitional section, where the root diameter gradually decreases from 23.8 mm to 17.9 mm. In Region C, the root diameter remains constant at 17.9 mm. The major (outer) diameter is uniformly 28 mm across all three regions, and the thread pitch is 28 mm.

**Figure 11:**
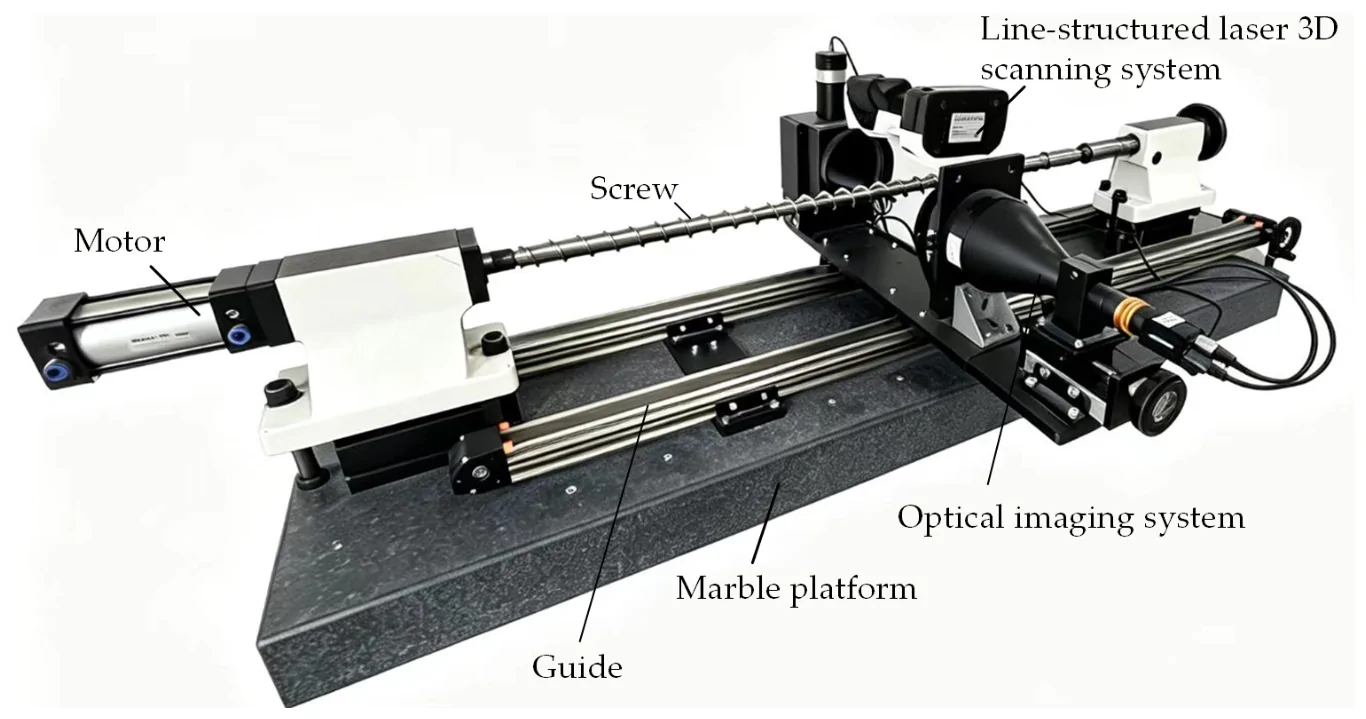
Experimental setup for the collaborative laser-vision measurement system.

The experimental setup is illustrated in Figure 11. The tested screw thread is securely clamped between two centers and can be rotated about its axis via a rotary motor. An imaging system mounted on a linear guide rail moves along the axial direction of the screw thread for data acquisition.

During the inspection procedure, the line-structured laser scanner first captures the initial three-dimensional point cloud of the screw thread, while the vision system simultaneously acquires two-dimensional images. Subsequently, the proposed vision-guided refinement method is applied to refine the thread edge contours extracted from the images. The refined edge contours are then re-projected onto the three-dimensional point cloud to correct the spatial positions of the edge points, thereby achieving precise alignment between the vision-based edge information and the laser point cloud data.

The key specifications of the measurement system are summarized in Table 3. The optical imaging system consists of a lens (Komavision Corporation, Dongguan, China) with a magnification of 0.13× and a camera (Hikvision Corporation, Hangzhou, China) with a pixel size of 3.45 μm and an image resolution of 2448 × 2048 pixels. The line-structured laser scanning system (Scantech, Corporation, Hangzhou, China) provides a laser scanning accuracy of 0.1 mm and a laser stitching accuracy of 0.3 mm/m. It achieves a maximum point acquisition rate of 1.5 × 10^6^ points/s, enabling the rapid acquisition of dense, high-quality point cloud data for accurate geometric characterization of helical surfaces. All algorithms were implemented in MATLAB R2023a and executed on a laptop with a 13th Gen Intel Core i9-13900HX CPU (Intel Corporation, Santa Clara, CA, USA), 16 GB of RAM, and an NVIDIA GeForce RTX 4060 GPU 8 GB (NVIDIA Corporation, Santa Clara, CA, USA).

**Figure 12:**
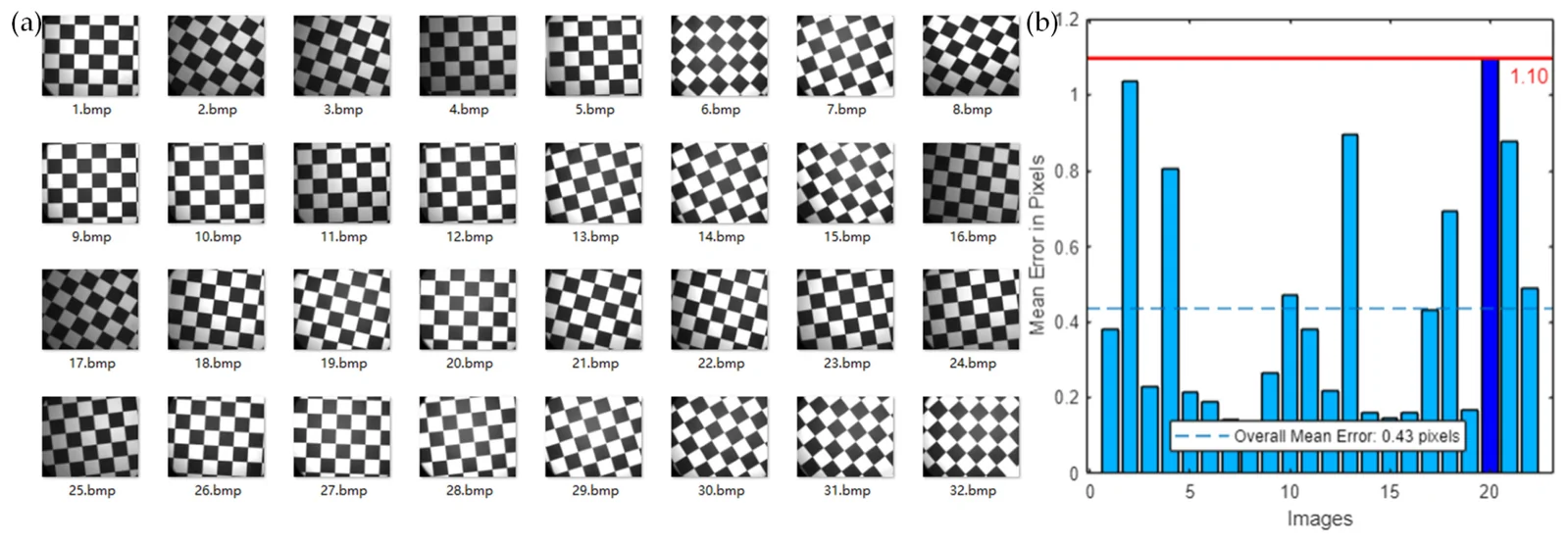
(**a**) Checkerboard images collected by imaging system, (**b**) reprojection errors of each checkboard image.

A checkerboard pattern with a minimum square side length of 10 mm was used for calibration. The calibration procedure was performed using the 32 checkerboard images illustrated in Figure 12a. Among them, 10 images with excessive reprojection errors were discarded after quality screening. The reprojection errors for remaining calibration images are summarized in Figure 12b, with the mean reprojection error calculated as 0.43 pixels (the joint calibration error was 2.45 pixels). A search radius of 3 pixels depicted in Section 2.4 is selected. Since it is slightly larger than this combined two errors, providing a sufficient margin for residual calibration errors and synchronization errors.

**Figure 13:**
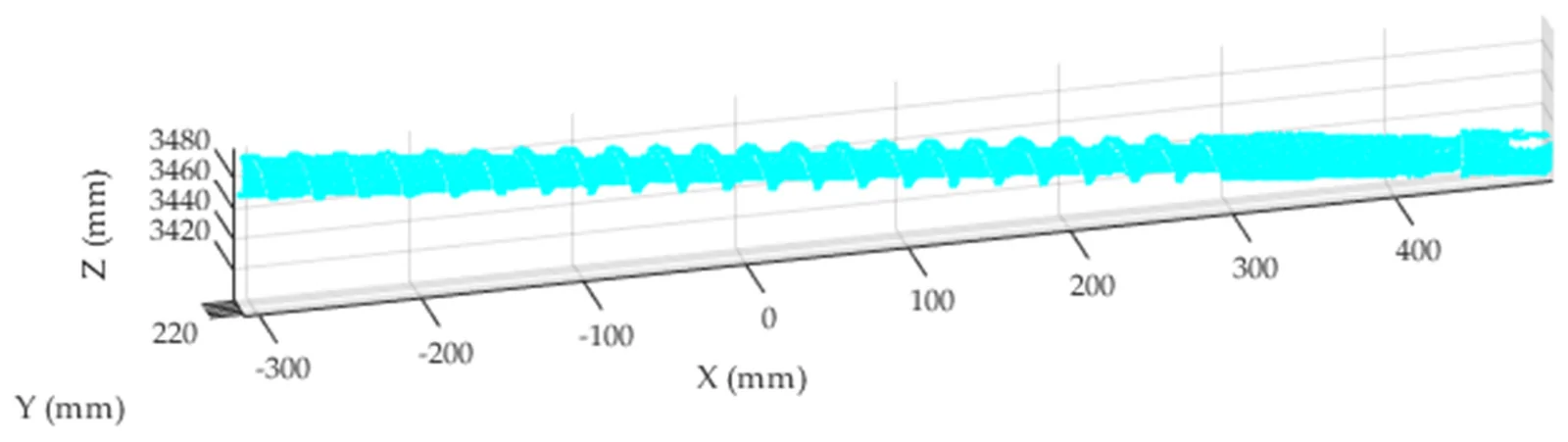
Three−dimensional point clouds of the tested screw thread.

**Figure 14:**
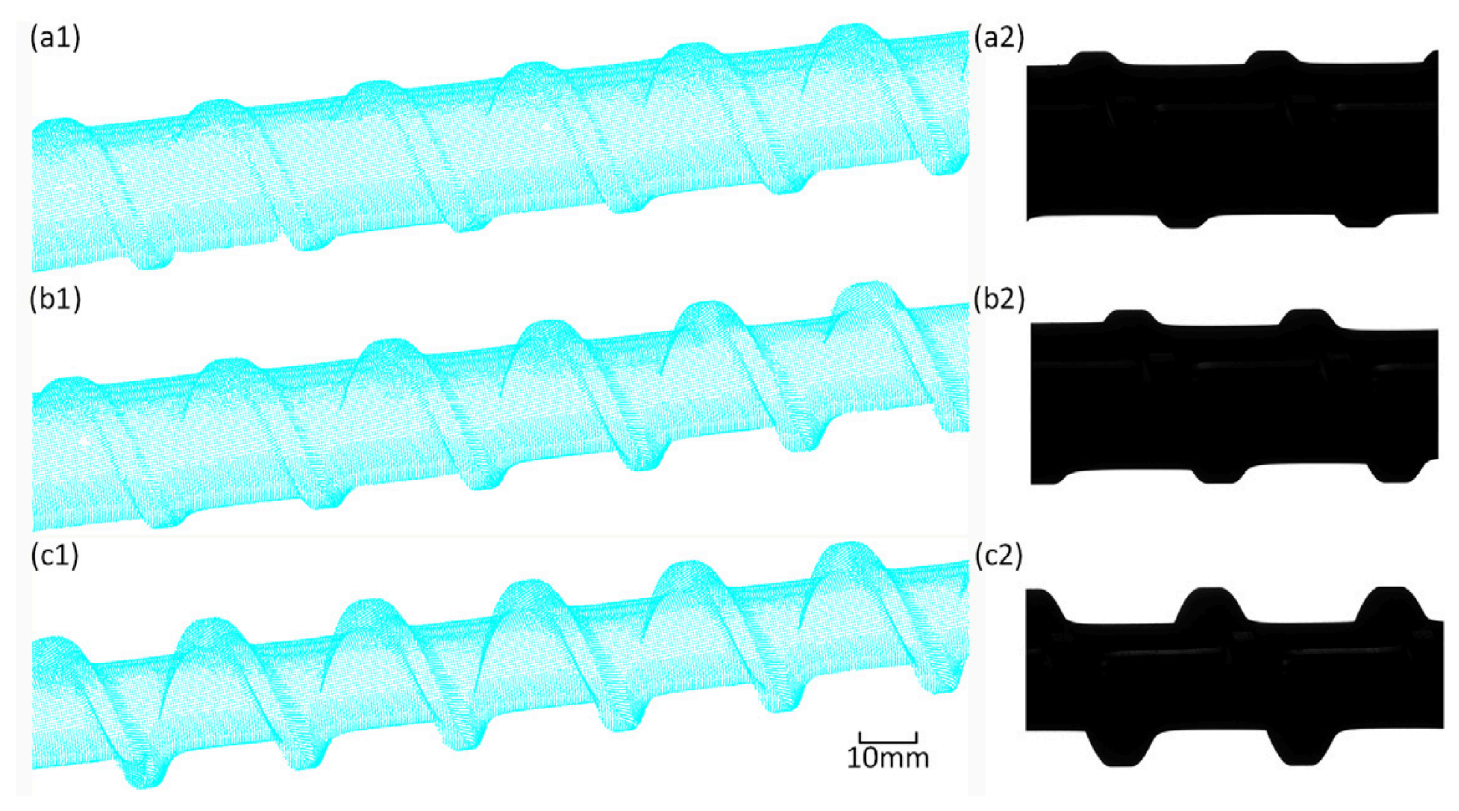
Three-dimensional point clouds (**a1**–**c1**) and corresponding vision−based thread images (**a2**–**c2**) of the tested screw thread in regions A, B, and C as defined in Figure 10.

The three-dimensional point cloud data corresponding to the specimen shown in Figure 10 are presented in Figure 13. The localized magnified views of regions A, B, and C (as marked in Figure 10) are shown in Figure 14(a1,b1,c1), respectively, while the corresponding images captured by the vision system are displayed in Figure 14(a2,b2,c2). Specifically, Figure 14(a1,a2) correspond to region A, where the nominal root diameter is 23.8 mm. Figure 14(b1,b2) correspond to the transitional region B, where the root diameter gradually tapers from 23.8 mm to 17.9 mm. Figure 14(c1,c2) correspond to region C, where the nominal root diameter is 17.9 mm.

#### 3.2.2. Validation of Vision-Guided Point Cloud Correction

**Figure 15:**
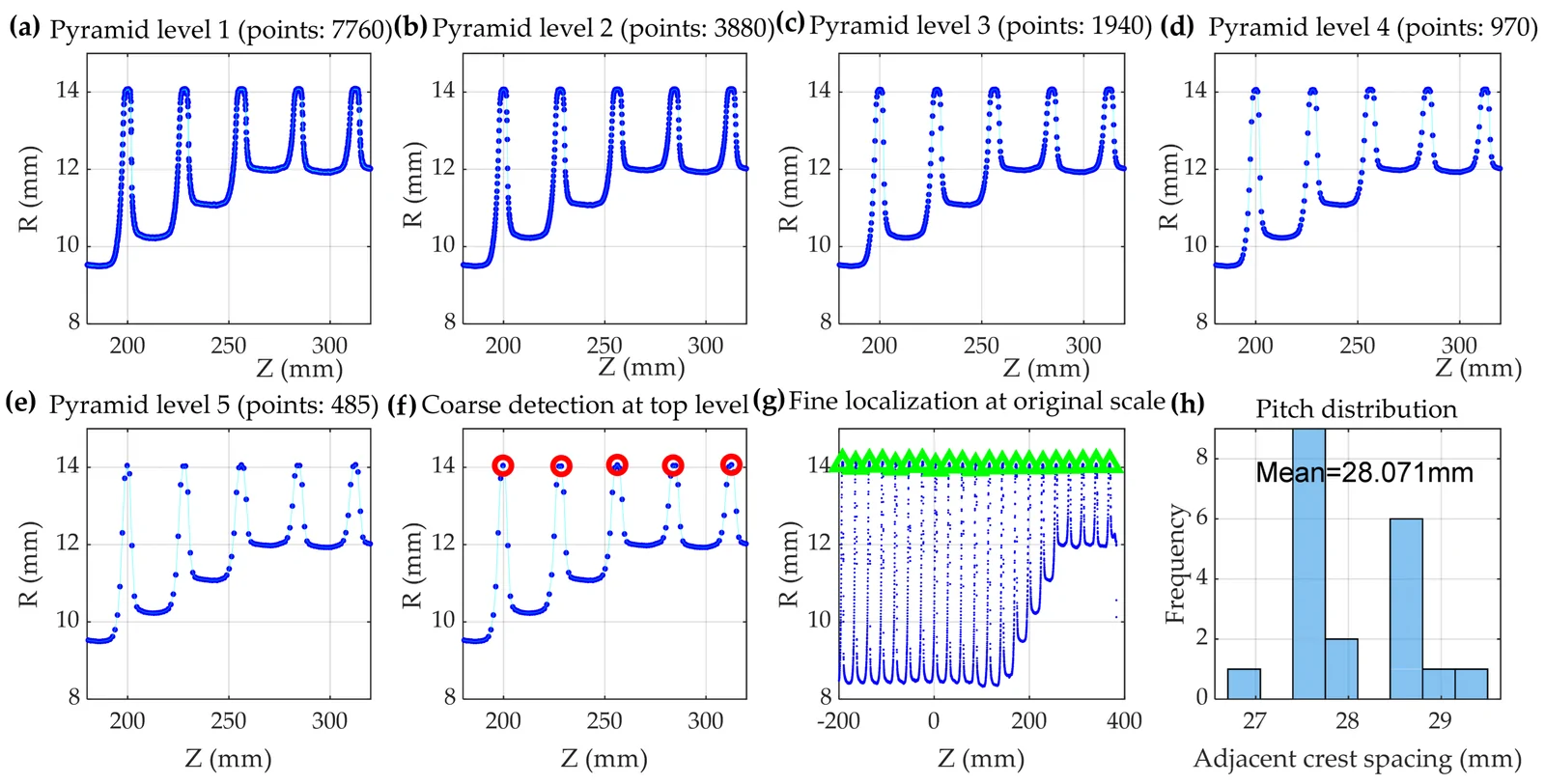
Experimental results of the proposed vision−guided point cloud refinement method for one section. (**a**–**e**) Pyramid layers 1 to 5 (from original to coarsest scale). (**f**) Coarse crest detection at the top level. (**g**) Fine crest localization at the original scale. (**h**) Histogram of local pitch distribution.

The methods in Section 2 were applied to the data in Figure 13. For illustration, Figure 15 shows the result from one representative longitudinal section. A five-level Gaussian pyramid was used, as determined by Equation (14).

The point counts at Levels 1, 2, 3, 4 and 5 are 7760, 3880, 1940, 970 and 485, respectively, as shown in Figure 15a–e. At the coarsest level (Level 5), the algorithm detected 21 candidate crest positions through coarse localization, as presented in Figure 15f. Subsequently, these coarse positions were mapped back to the high-resolution data at Level 1, where fine localization was performed by accurately determining the true crest positions in the vicinity of each candidate point, as illustrated in Figure 15g. Based on the refined crest positions, the mean pitch of one section was estimated to be 28.071 mm, with the pitch distribution shown in Figure 15h. To evaluate the stability of the multi-section fusion, the pitch was estimated independently from each of the nine longitudinal sections. The nine section-wise pitch values range from 28.056 mm to 28.075 mm, with a mean of 28.064 mm and a standard deviation of 0.014 mm.

Table 4 presents a comparative evaluation of the proposed method against alternative approaches for thread pitch measurement, with the CMM result serving as the reference. The CMM reference measurement was performed using a Zeiss PRISMO ultra coordinate measuring machine No. 197409 (ZEISS Group, Oberkochen, Germany). Its uncertainty is *U* = (0.16 + 0.8 *L*/1000) μm and coverage factor *k* = 2 (Metrology Certificate No. XZJH-20260151132), which is recently calibrated by Zhejiang Institute of Quality Sciences (China National Accreditation Service for Conformity Assessment Laboratory Accreditation Certificate Registration No. CNAS L2865). Ten repeated measurements were performed on each of the three regions (A, B, C) of the thread workpiece. The pitch was determined as the mean of these ten measurements, yielding a pitch value of 28.0217 mm with a standard deviation of 0.033 μm, which is adopted as the reference value.

The vision-based method alone, utilizing 21,080 points at the final profile, produces a pitch of 27.9715 mm with an absolute error of 0.0502 mm and a processing time of 1.56 s. This indicates that purely image-based measurement can provide a reasonable estimate, albeit at the cost of a large number of points and a relatively long processing time.

The Fourier transform-based method, employing 7760 points at the final profile, yields a pitch of 25.9 mm, corresponding to an absolute error of 2.1217 mm with a processing time of 0.72 s. This large deviation suggests that frequency-domain analysis alone, without subsequent refinement, is inadequate for high-precision pitch measurement. By incorporating Gaussian pyramid multi-resolution refinement, the error is significantly reduced from 2.1217 mm to 0.0783 mm, despite a 94.7% reduction in the number of points at the final profile (from 7760 to 412). The processing time remains comparable (0.74 s). This substantial improvement validates the effectiveness of the coarse-to-fine refinement strategy in enhancing measurement accuracy while simultaneously reducing data redundancy.

The proposed method, which further incorporates vision-guided point cloud refinement, achieves a pitch of 28.064 mm, with an absolute error of 0.0423 mm and a processing time of 0.81 s. This represents a marked improvement over the Fourier transform-only method and achieves an accuracy comparable to the pure vision-based method, but with substantially fewer points (485 vs. 21,080) and a much shorter processing time (0.81 s vs. 1.56 s). Notably, compared with the Fourier transform + Gaussian pyramid method, the proposed method reduces the absolute error by approximately 46% (from 0.0783 mm to 0.0423 mm), demonstrating the additional benefit of vision-guided point cloud correction.

In terms of computational efficiency, the proposed method requires 0.81 s per measurement, which is comparable to the Fourier transform + Gaussian pyramid method (0.74 s) and significantly faster than the pure vision method (1.56 s). The processing time of 0.81 s refers to the complete per-measurement pipeline, which includes PCA-based principal axis alignment, vision-guided point cloud correction, FFT-based coarse pitch estimation, and Gaussian pyramid refinement. It should be noted that the one-time calibration of the camera and the laser-camera extrinsic parameters is not included, as these are performed only once during system setup and remain fixed for subsequent measurements.

This demonstrates that the proposed method achieves a favorable balance between computational efficiency and measurement accuracy, making it suitable for practical industrial inspection tasks where both precision and throughput are critical.

Overall, these results confirm that vision-guided point cloud refinement effectively enhances the measurement accuracy of the laser scanning system, with the coarse-to-fine strategy playing a crucial role in the refinement pipeline. The proposed method offers a balanced trade-off between accuracy and computational cost, underscoring its potential for high-precision screw thread inspection in real-world manufacturing environments.

## 4. Conclusions

This paper presented a vision-guided point cloud refinement method for screw thread pitch measurement using line-structured laser scanning. Sub-pixel edge positions are extracted from camera images by a sub-pixel localization and interpolation algorithm. Then, these positions are used to directly correct the spatial coordinates of thread crest points in the laser point cloud. It transfers the high lateral resolution of machine vision directly to the 3D measurement domain.

In simulations, PCA alignment achieved a mean angular error of 0.078° across 8281 poses, and Gaussian pyramid refinement reduced pitch error from 7.57% to 0.28%, reducing final profile points from 7760 to 412. In real experiments, the method achieved a pitch of 28.064 mm with an absolute error of 0.0423 mm relative to the CMM reference (28.0217 mm). Sensitivity analysis showed robustness to noise and outliers (up to 10%), but higher sensitivity to missing data.

Limitations include validation on a single specimen under laboratory conditions, performance degradation when more than 20% data are missing, focus only on pitch, and reliance on accurate camera-laser calibration. In addition, the PCA-based principal axis alignment is highly sensitive to circumferential coverage. The angular error increases sharply when the coverage falls below 270°. This means the method may not be reliable for workpieces that are only partially scanned.

Future work will extend validation to more thread specifications, materials, and real manufacturing environments, and to other geometric parameters such as major diameter, root diameter, lead, and flank angle. Measurement uncertainty will be evaluated following metrological guidelines. Overall, the method shows promise for high-precision thread pitch measurement by transferring sub-pixel vision precision to the 3D point cloud domain.

## Figures and Tables

**Table 1:** Basic numerical setting parameters.

Canny Double Thresholds	Kernel Size of Gaussian Filter for Image	Kernel Size of Median Filter for Edge Contour	Kernel Size of Gaussian Filter for Edge Contour	Outlier Rejection Threshold
50 (low), 170 (high)	5 × 5	1 × 11	1 × 7	3

**Table 2:** Specifications of the simulation parameters.

Thread Profile	Number of Points	Screw Pitch	Thread Depth
Trapezoidal	4.74 × 10^5^	28 mm	2 mm

**Table 3:** Specifications of the measurement system.

Lens (KOMAVISION)	Camera (HIKVISION)		The Line-Structured Laser Scanning System (SCANTECH)		
Magnification	Pixel Size	Resolution	Laser Scanning Accuracy	Laser Stitching Accuracy	Maximum Point Acquisition Rate
0.13	3.45 μm	2448 × 2048	0.1 mm	0.3 mm/m	1.5 × 10^6^ points/s

**Table 4:** Measurement errors of different methods relative to the CMM reference.

Method	Number of Points at Final Profile	Processing Time from Original Profile to Final Profile(s)	Measured Pitch (mm)	Absolute Error (mm)
Coordinate measuring machine (CMM)	-	-	28.0217	-
Vision image	21,080	1.56	27.9715	0.0502
Fourier transform	7760	0.72	25.9	2.1217
Fourier transform + Gaussian pyramid	412	0.74	28.1	0.0783
Proposed method	485	0.81 (excludes one-time calibration)	28.064	0.0423

## Data Availability

The original contributions presented in this study are included in the article. Further inquiries can be directed to the corresponding author.
